# Investigation of Super Learner Methodology on HIV-1 Small Sample: Application on Jaguar Trial Data

**DOI:** 10.1155/2012/478467

**Published:** 2012-04-03

**Authors:** Allal Houssaïni, Lambert Assoumou, Anne Geneviève Marcelin, Jean Michel Molina, Vincent Calvez, Philippe Flandre

**Affiliations:** ^1^INSERM, UMR-S 943, 56 Boulevard Vincent Auriol, BP 335, 75625 Paris Cedex 13, France; ^2^UPMC Univ Paris 06, UMR S943, Paris, France; ^3^Service de Virologie, Hôpital Pitié-Salpêtrière, AP-HP, Paris, France; ^4^Service des Maladies Infectieuses, Hôpital Saint Louis, AP-HP, Paris, France

## Abstract

*Background*. Many statistical models have been tested to predict phenotypic or virological response from genotypic data. A statistical framework called Super Learner has been introduced either to compare different methods/learners (discrete Super Learner) or to combine them in a Super Learner prediction method. *Methods*. The Jaguar trial is used to apply the Super Learner framework. The Jaguar study is an “add-on” trial comparing the efficacy of adding didanosine to an on-going failing regimen. Our aim was also to investigate the impact on the use of different cross-validation strategies and different loss functions. Four different repartitions between training set and validations set were tested through two loss functions. Six statistical methods were compared. We assess performance by evaluating *R* ^2^ values and accuracy by calculating the rates of patients being correctly classified. *Results*. Our results indicated that the more recent Super Learner methodology of building a new predictor based on a weighted combination of different methods/learners provided good performance. A simple linear model provided similar results to those of this new predictor. Slight discrepancy arises between the two loss functions investigated, and slight difference arises also between results based on cross-validated risks and results from full dataset. The Super Learner methodology and linear model provided around 80% of patients correctly classified. The difference between the lower and higher rates is around 10 percent. The number of mutations retained in different learners also varys from one to 41. *Conclusions*. The more recent Super Learner methodology combining the prediction of many learners provided good performance on our small dataset.

## 1. Introduction

The effectiveness of antiretroviral therapy has been limited by the development of human immunodeficiency virus type 1 (HIV-1) drug resistance. HIV-1 frequently develops resistance to the antiretroviral drugs used to treat it which may decrease both the magnitude and the duration of the response to treatment resulting in loss of viral suppression and therapeutic failure [1]. Moreover, there is a high level of cross-resistance within drug classes; a virus that has developed resistance to one drug in a class may also be resistant to other drugs in the same class [2]. Current International AIDS Society USA and French report HIV-1 guidelines recommend resistance testing both before starting antiretroviral therapy (ART) and at treatment failure. Resistance testing has become an important part of choosing and optimizing combination therapy for treating HIV-infected individuals [3]. Selecting a “salvage” regimen for an HIV-infected patient who has developed resistance to his or her current regimen is not straightforward [4].

Genotypic or phenotypic assays are used for resistance testing each, assay having advantages and limitations. From those assays we used either the genotypic-phenotypic correlation, showing phenotypic effect of mutations, or the genotypic-virologic correlation, investigating the impact of mutations on the virological response to a subsequent treatment. The latter correlation is mainly used by the *Agence Nationale de Recherches sur le SIDA* to build rule-based algorithms (ANRS http://www.hivfrenchresistance.org/). The increasing number of antiretroviral drug-resistance-associated mutations has increased the difficulty of the interpretation of those assays [5].

In both cases many HIV-1 drug resistance analysis approaches have been explored, from simple linear models [6] to more sophisticated ones, such as database pattern search method [7], neural networks/machine learning [8–11], or genotype-phenotype mapping [12]. Such methods, or learners, differ by the mechanism used to search over the space of parameters. It appears that different interpretation systems lead to distinct results [13–15]. Current widely used genotypic interpretation systems may have no satisfactory performance on newly derived datasets. Such poor performances emphasize the need for an external validation dataset or a sufficient large database to create a validation set. It has been shown that the variability observed in different rule-based algorithms was mainly due to the patients' baseline characteristics than to the statistical methods used [16, 17].

A framework for the unified loss-based estimation suggested a solution to this problem in the form of a new estimator, called the “Super Learner” [18, 19]. Initially this methodology, called Discrete Super Learner, compared different learners (methods) on the basis of the loss-based estimation theory and choose the optimal learner for a given prediction problem based on cross-validated risk (repartition between training sample and validation sample) [20]. The Super Learner methodology has been improved building now an estimator based on a linear combination of the different learners investigated [19, 21, 22].

Originally, the Super Learner used both mean square of residuals (differences between observed and predicted outcomes) and *R* ^2^ for evaluation and assessment. However, statistical investigations showed the importance of exploring different loss functions [23], such as first-order coefficient *R*.

Our aim is to study the performance of the discrete and the most recent Super Learner methodology on a small sample of HIV-1 data from a randomized clinical trial. Especially, based on this methodology, we investigate four different cross-validation setting, and the use of two loss functions for six statistical learning methods. This methodology is applied on the Jaguar trial data [24].

## 2. Methods

### 2.1. Datasets

For a patient *i*, the data consist of a vector *X*_*i*_ of binary variables indicating presence or absence of a mutation and *Y*_*i*_ denotes the virologic outcome. In the regression setting, the objective is to predict *Y* using *X*. Then, the parameter of interest is denoted as *E*(*Y* | *X*). We analyzed the data obtained from the Jaguar trial which are described elsewhere [24]. Briefly the Jaguar trial was a randomized multicenter, double-blind placebo-controlled trial evaluating the efficacy of adding didanosine (ddI) to an on-going antiretroviral (ARV) regimen. Patients were randomly assigned at a ratio 2 : 1 to receive ddI or a matching placebo added to their current regimen. The primary efficacy end point was the magnitude of change in plasma HIV-1 RNA levels in log⁡_10_ copies per mL from baseline to week 4. The naïve method was used to compute viral load reduction; that is, all HIV-1 RNA levels <50 copies/mL at week four were fixed at 50 copies/mL. Although censored methods are preferred to compute HIV-1 RNA changes, the low percentage (11%) of patients censored provides in this case an unbiased estimate [25–27]. The median changes in HIV-1 RNA at week 4 were −0.56log⁡_10_ copies/mL (IQR, −0.14 to −1.2) and +0.07log⁡_10_ copies/mL (IQR, 0.12 to 0.21) in patients receiving ddI and placebo, respectively (*P* < .0001). HIV-1 sequences were available for all patients, but only patients in the ddI group were used in the present work. HIV-1 sequences and HIV-1 RNA reduction at week 4 were available for 102 patients. Mutations were defined as amino acid differences from subtype B consensus wild-type sequence (wild-type virus HXB2). We investigate the virologic impact at week 4 of ten resistance mutations: M41L (prevalence 48%), D67N (34.3%), T69D (8.8%), K70R (26.5%), L74V (8.8%), V118I (18.6%), M184VI (92.2%), L210W (27.5%), T215Y/F (53.9%), and K219Q/E (24.5%). This set has been the starting point for building ANRS ddI rules and was potentially linked to the ddI resistance at the time of the study. Moreover, the choice of using a subset of mutations is driven by Soo Yon Rhee et al. study [28], in which they show that expert mutation selection is preferable than using the entire sequences.

### 2.2. Super Learner

The methodology has been proposed by Mark van der Laan et al. [18, 19] as a setting to choose the optimal learner (method) among a set of candidate learners, this version of the methodology was called the Discrete Super Learner. Recently, the methodology has been refined and proposed a new estimator based on a weighted linear combination of candidate learners to build a Super Learner estimator [19, 21, 22]. We briefly introduced the general principle and few key features of this methodology. The general strategy for loss-based estimation is driven by the choice of a loss function and relies on cross-validation for estimator selection and performance assessment. Cross-validation divides the available dataset into *k* mutually exclusive and exhaustive sets of as nearly equal size as possible. Each set and its complement play the role of the validation and training samples. Observations in the training set are used to construct (or train) the estimators, and observations in the validation set are used to assess the performance (or validate) of the estimators. For each estimator/learner the *k* risks over the *k* validation sets are averaged resulting in the so-called cross-validated risk. For example, with a 10-fold cross-validation the learning set is partitioned into 10 parts, each part in turn served as a validation set, while the other 9/10ths of the data served as the training set. Based on cross-validated risks, estimators/learners can be ranked from those identified as top learners to those providing poor performance. In the discrete version of the methodology, the optimal learner is applied to the entire dataset. In the most recent version, a new estimator (the Super Learner) is proposed based on a family of weighted combinations of the estimators/learners. The new Super Learner appears as a generalization of the discrete Super Learner.

We applied all individual learners and the new estimator on full dataset (which will be called full model in the following). Learners are ranked from those identified as top learners to those providing poor performance. We investigate four splits: 10-fold, 4-fold, 3-fold, and 2-fold that correspond to 90%, 75%, 66%, and 50% of data use as training samples and 10%, 25%, 33%, and 50% as validation sample respectively. Learners were evaluated using two distinct functions usually used as loss functions: squared error (SqE) and first-order coefficient (*R*). The SqE is (*Y*−*E*(*YX*))^2^, that is, the squared difference between observed and predicted outcome. *R* is the first-order correlation coefficient between *Y* and *E*(*Y* | *X*), which has been recently used in this context [29]. It is important to note that SqE is unbounded while −1 ≤ *R* ≤ 1. For all full models, *R* ^2^ estimates and accuracy were also computed in addition to SqE and *R*.

We defined two threshold values to define patients having a virologic response: −0.6log⁡_10_ copies/mL and −0.5log⁡_10_ copies/mL. For example, a patient with an HIV-1 RNA reduction larger than 0.6log⁡_10_ copies/mL was classified as responder, otherwise as nonresponder. Patients may also be classified responders or not according to the predicted reduction by a given method.

## 3. Candidate Learners

We investigate the following learners: Logic Regression, Deletion/Substitution/Addition, Least squares regression, Random Forest, Classification and Regression Trees. All algorithms are available as free packages of *R* software.

Logic Regression (package named *LogicReg*) is an adaptive regression methodology that attempts to construct predictors as Boolean combinations of covariables [30]. Deletion/Substitution/Addition (package named *DSA*) is polynomial regression dataadaptive that generates candidate predictors as polynomial combinations of binary covariables [31]. Classification and Regression Trees (CARTs) build a regression tree in continuous outcome setting (package *rpart*) [32]. Random Forest (package *RandomForest*) is a “bagging predictor” (Bootstrap Aggregating), this method build a model from a combination of high number of regression trees resulting in the so-called Forest [33]. Least squares regression was set up on two datasets: one consisted of all main terms and the second consisted of all main terms plus all two-way interactions (resp. denoted as LM(1) and LM(2)).

From those learners, we set up two Super Learners: Super Learner using five learners, built with D/S/A, LM(1), LM(2), random forest and CART (noted Super Learner-5 in the following), and Super Learner with six learners, the same as Super Learner-5 plus Logic Regression (denoted as Super Learner-6 in the following).

Internal fine-tuning procedure by internal cross-validation was used to obtain the best performance for Logic Regression and D/S/A. The tuning parameters of D/S/A were *maxsize* = *20* (two times the number of co-variables), *maxorderint* = *2* and *maxsumofpow* = *2*. All three steps were allowed (*Deletion*, *Substitution,* and *Addition*). CART has *complexity parameter* (*cp*) equal to 0.01. For Random Forest the number of trees was *1,000* and the number of variables to randomly consider at each node of each tree was fixed at three (*m*_try_ = 3). That corresponds to the number of co-variables divided by 3 which is usually used in regression setting. Simple linear regression was used as reference (without variable selection procedure). Methods were ranked; if two or more methods produced the same risk value, the mean rank was assigned (e.g., if Super Learner-5 and LM (1) gave the same SqE, in spite of assigning rank 1 and 2, resp., we noted 1.5 for both).

## 4. Results

Results of the Discrete Super Learner and Super Learner-5 are given in Table 1. For example, based on the SqE as loss function and a 10-fold cross-validation, LM(1) was identified as the top learner followed by Random Forest and CART. LM(1) slightly decreases its performance from the 1st rank on 10-fold to 3th rank on 2-fold while Random Forest becomes the second learners for the remaining *k*-folds. Surprisingly, linear model with interaction terms, LM(2), provided poor performance for all *k*-fold. The Super Learner-5 provided at least as good performance as the top learner whatever the *k*-fold cross-validation. *R* loss function drew similar findings. Although the ranks of the different learners are relatively stable, the combination of the Super Learner-5 provided the best performance. Inclusion of Logic Reg as additional learner in the previous set of candidate learners led to different findings (Table 2). Globally Logic Reg performed poorly, and only LM(2) produced worse performance than Logic Reg. Based on the SqE as loss function, including Logic Reg in the Super Learner-6 decreased its performance compared to Super Learner-5. Based on *R* as loss function, the performance of the Super Learner-6 was very good.

**Figure 1:**
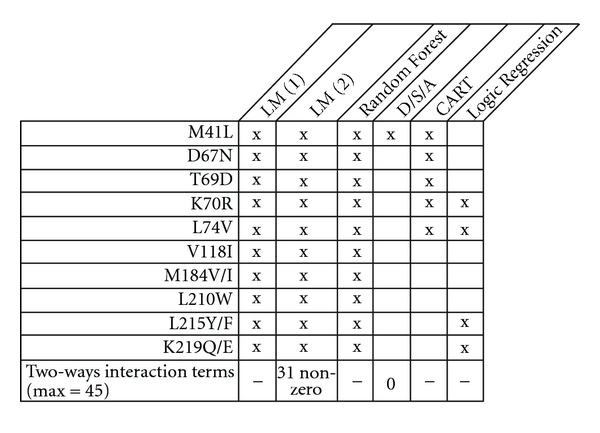
Selected mutations for each model on the complete Jaguar data Trial.

We applied all learners including Super Learner-5 and Super Learner-6 on the entire dataset (Table 3). Based on SqE, *R*, and *R* ^2^ measure estimates, Super Learner-5 and −6 provided very good performances. The use of LM(2) on the full dataset provided a high level of prediction (*R* ^2^ = 0.540) while, based on *k*-fold cross-validated risk, this learner was the poorest candidate. Comparing cross-validation and full model results indicate the LM(2) model was over fit.  Figure 1 displays the mutations retained by each learner. All mutations were retained for LM(1), LM(2), and Random Forest (not surprisingly all mutations are at least selected one time in a tree). CART selected M41L, D67N, T69D, K70R, L74V, and K219Q/E mutations. Of note the D/S/A method selected only the M41L mutation which should be balanced with its poor performance.

**Figure 2:**
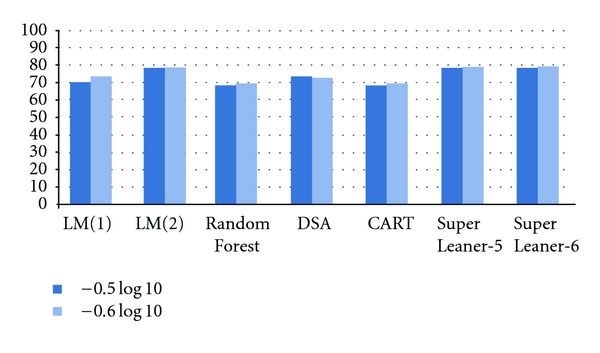
Rates of patients being well classified for threshold −0.5 and −0.6log⁡_10_ for all models applied on the Jaguar trial full-dataset.

The final goal of interpreting genotypic resistance testing is to classify patients as “sensitive” or “resistant” to a specific drug.  Figure 2 displays the rates of patients being well classified for the two threshold values investigated. For both threshold values LM(2), Super Learner-5 and −6 have the highest accuracy with around 80% of patients correctly classified. CART and Random Forest provided the lowest accuracy, slightly below 70% of patients correctly classified, corresponding to a 10% difference. As expected the accuracy of Random Forest model depends on the *m*_try_ values.

## 5. Discussion

The choice of subsequent treatment in failing patients is of major importance in the management of HIV-infected patients. Genotypic and phenotypic resistance tests are important tools for choosing promising combination therapy for those patients. We investigated on a small sample a framework both for choosing optimal learner and building an estimator among a set of candidate through two different loss functions and *k*-fold cross-validation.

Based on cross-validation risk, the Super Learner estimator was the “best” learner though the linear model with only main terms LM(1) providing similar performance to that of Super Learner-5 and -6. The use of the SqE as loss function indicated that the inclusion of Logic Reg as an additional learner decreased the performance of the Super Learner estimator. However, prediction results based on the full dataset as well as accuracy questioned the use of SqE as loss function, although it is known that full dataset provided different results than those based on cross-validation strategy [34, 35]. Based on cross-validation risk, the good performance of LM(1) should be compared with the poor performance of the linear model with interaction terms LM(2). Inversely, LM(2) outperforms LM(1) in the full dataset. In our small dataset, this finding is clearly due to overfit of the data by the LM(2) model. A researcher ignoring the Super Learner methodology using a linear model with interaction terms would obtain a good performance on the full dataset while such a learner would have not been selected from the discrete Super Learner methodology.

The choice of *m*_try_ parameter for Random Forest is a real problem. However, the common *m*_try_ used in regression setting (number of covariables divided by three) appears as a good compromise. Whatever the *m*_try_ value is, all mutations were selected at least on time using Random Forest on full dataset. This was expected due to the relative small number of mutations compared with 1,000 trees generated by the Random Forest model

The HIV-1 resistance study used either a continuous outcome (as HIV-1 RNA reduction from baseline to the time of interest) or a categorical outcome (classifying patients as achieving a virologic response at the time of interest). For example, virologic response can be defined an HIV-1 reduction of 1.5log⁡_10_ copies/mL or more or having a viral load >50 copies/mL at the time of interest. Even if a continuous outcome is preferable as being more informative, the final goal of determining the drug resistance mutations associated with a poorer virologic response is to classify patients as “sensible” or “resistant” to a specified drug. The former patients would receive the corresponding drug as a part of their regimen while the latter patients would not. We used two threshold values of −0.5 and −0.6log⁡_10_ copies/mL to define virologic response. For both threshold values LM(2), Super Learner-5 and -6 provided the highest accuracy with approximately 80% of patients correctly classified.

All the methods used in this work are usually applied to large or very large datasets. Simple linear regression model was fitted on more than 5,000 genotype-phenotype paired datasets from the same database [6]. Investigation of logistic regression and nonlinear machine learning for predicting response to antiretroviral treatment was done on more than 3,000 treatment change episodes from the EuResist database [34]. All these analyses were made retrospectively mainly for comparing different methods rather than for building rule-based algorithm.

A major reason to apply the Super Learner methodology on the Jaguar trial is that often the first version of an algorithm for a specific drug is based on a limited amount of data [35–37]. Such algorithms are updated later with publication of new data. Nonparametric methods are then often used on such a relative small amount of data [38, 39]. Parametric methods have the advantage of not only integrating two-way interactions terms but also adjusting for some other variables that improve the prediction. Randomized clinical trials, in treatment experienced patients, provide frequently the first opportunity to investigate the impact of baseline mutations in the subsequent virologic response in those patients. It was then of interest to know whether the Super Learner methodology applied only on around one hundred of patients was able to produce the “best” learner on the basis of accuracy and prediction. The Jaguar trial which is an “add-on” study ensuring a good quality of relation between reverse transcriptase mutations and effect on the drug investigated, was a good opportunity for such investigation.

It has been shown that, in the context of genotype-phenotype correlation with a large database, the linear model without interactions provided also accurate predictions [6]. However, based on the full dataset results, we highlight the importance of the two-way interactions terms for Least Squares. Interactions between mutations are of scientific interest, both to help in drug selection and to understand mechanisms of resistance.

## 6. Conclusion

In this study, we showed that the Super Learner methodology applied on a relative small amount of data, provided good performance. Of note in our dataset, simple linear regression with two-way interaction terms performs as well as the Super Learner.

##  Author's Contribution

A. Houssaïni and P. Flandre designed research; A. Houssaïni and P. Flandre performed analysis; A. Houssaïni, L. Assoumou, A. G. Marcelin, J. M. Molina, V. Calvez and P. Flandre discussed the results and improved the paper.

## Figures and Tables

**Table 1:** Squared error, *R* detailed values and corresponding rank on Jaguar trial data for 10-fold, 4-fold, 3-fold, and 2-fold.

SqE without Logic Reg.									

	10-fold		4-fold		3-fold		2-fold		
Method	Rank	Mean	Rank	Mean	Rank	Mean	Rank	Mean	Mean rank

LM(1)	1.5	0.216	3	0.246	3	0.238	3	0.293	2.625
LM(2)	6	1.218	6	1.267	6	1.650	6	1.117	6
Random Forest	3	0.258	2	0.241	2	0.235	2	0.275	2.25
D/S/A	5	0.283	4	0.264	4	0.255	4	0.295	4.25
CART	4	0.264	5	0.267	5	0.258	5	0.298	4.75
Super Learner-5	1.5	0.216	1	0.238	1	0.228	1	0.273	1.125

1 − *R* without Logic Reg.									

	10-fold		4-fold		3-fold		2-fold		
Method	Rank	Mean	Rank	Mean	Rank	Mean	Rank	Mean	Mean rank

LM(1)	1.5	0.464	3	0.554	3	0.534	1.5	0.651	2.25
LM(2)	6	0.808	6	0.724	6	0.686	6	0.754	6
Random Forest	3	0.609	2	0.552	2	0.532	3	0.656	2.5
D/S/A	5	0.712	4	0.623	4	0.607	5	0.746	4.5
CART	4	0.632	5	0.644	5	0.611	4	0.743	4.5
Super Learner-5	1.5	0.464	1	0.539	1	0.508	1.5	0.651	1.25

**Table 2:** Squared error, *R* detailed values and corresponding rank on Jaguar trial data for 10-fold, 4-fold, 3-fold, and 2-fold.

SqE with Logic Reg.									

	10-fold		4-fold		3-fold		2-fold		
Method	Rank	Mean	Rank	Mean	Rank	Mean	Rank	Mean	Mean rank

LM(1)	1	0.216	2	0.246	2	0.238	2	0.293	1.75
LM(2)	7	1.218	7	1.267	7	1.650	7	1.117	7
Random Forest	2	0.258	1	0.241	1	0.235	1	0.275	1.25
D/S/A	4	0.283	3	0.264	3	0.255	3	0.295	3.25
CART	3	0.264	4	0.267	4	0.258	4	0.298	3.75
LogicReg	6	0.653	6	0.65	6	0.652	6	0.653	6
Super Learner-6	5	0.378	5	0.455	5	0.499	5	0.527	5

1 − *R* with Logic Reg.									

	10-fold		4-fold		3-fold		2-fold		
Method	Rank	Mean	Rank	Mean	Rank	Mean	Rank	Mean	Mean rank

LM(1)	1.5	0.464	3	0.554	3	0.534	2	0.651	2.375
LM(2)	7	0.808	7	0.724	7	0.686	7	0.754	7
Random Forest	3	0.609	2	0.552	2	0.532	3	0.656	2.5
D/S/A	6	0.712	4	0.623	4	0.607	6	0.746	5
CART	4	0.632	5	0.644	5	0.611	5	0.743	4.75
LogicReg	5	0.702	6	0.685	6	0.684	4	0.657	5.25
Super Learner-6	1.5	0.456	1	0.523	1	0.485	1	0.593	1.125

**Table 3:** Squared Error, *R*, *R* ^2^ and corresponding rank on Jaguar Trial full-dataset.

Full Model	SqE		1 − *R*/100		*R* ^2^	

Method	Rank	Value	Rank	Value	Rank	Value
LM (1)	5	0.204	5	0.435	4	0.319
LM (2)	1.5	0.138	1.5	0.265	1.5	0.540
Random Forest	4	0.178	4	0.348	6	0.271
D/S/A	7	0.242	7	0.561	7	0.193
CART	6	0.211	6	0.454	5	0.299
Super Learner-5	1.5	0.138	1.5	0.265	1.5	0.540
Super Learner-6	3	0.139	3	0.266	3	0.539

## References

[1] Lorenzi P, Opravil M, Hirschel B (1999). Impact of drug resistance mutations on virologic response to salvage therapy. *AIDS*.

[2] Palmer S, Shafer RW, Merigan TC (1999). Highly drug-resistant HIV-1 clinical isolates are cross-resistant to many antiretroviral compounds in current clinical development. *AIDS*.

[3] Aslanzadeh J (2002). HIV resistance testing: an update. *Annals of Clinical and Laboratory Science*.

[4] Costagliola D, Descamps D, Assoumou L (2007). Prevalence of HIV-1 drug resistance in treated patients: a French nationwide study. *Journal of Acquired Immune Deficiency Syndromes*.

[5] Shafer RW, Kantor R, Gonzales MJ (2000). The genetic basis of HIV-1 resistance to reverse transcriptase and protease inhibitors. *AIDS Reviews*.

[6] Wang K, Jenwitheesuk E, Samudrala R, Mittler JE (2004). Simple linear model provides highly accurate genotypic predictions of HIV-1 drug resistance. *Antiviral Therapy*.

[7] Kantor R, Machekano R, Gonzales MJ, Dupnik K, Schapiro JM, Shafer RW (2001). Human immunodeficiency virus reverse transcriptase and protease sequence database: an expanded data model integrating natural language text and sequence analysis programs. *Nucleic Acids Research*.

[8] Beerenwinkel N, Schmidt B, Walter H (2002). Quantitative phenotype prediction by support vector machines. *Antiviral Therapy*.

[9] Wang D, DeGruttola V, Hammer S (2002). A collaborative HIV resistance response database initiative: predicting virological response using neural network models. *Antiviral Therapy*.

[10] Sevin AD, DeGruttola V, Nijhuis M (2000). Methods for investigation of the relationship between drug-susceptibility phenotype and human immunodeficiency virus type 1 genotype with applications to AIDS Clinical Trials Group 333. *Journal of Infectious Diseases*.

[11] Drǎghici S, Potter RB (2003). Predicting HIV drug resistance with neural networks. *Bioinformatics*.

[12] Beerenwinkel N, Knupfer P, Tresch A (2011). Learning monotonic genotype-phenotype maps. *Statistical Applications in Genetics and Molecular Biology*.

[13] Assoumou L, Brun-Vézinet F, Cozzi-Lepri A (2008). Initiatives for developing and comparing genotype interpretation systems: external validation of existing systems for didanosine against virological response. *Journal of Infectious Diseases*.

[14] Ravela J, Betts BJ, Brun-Vézinet F (2003). HIV-1 protease and reverse transcriptase mutation patterns responsible for discordances between genotypic drug resistance interpretation algorithms. *Journal of Acquired Immune Deficiency Syndromes*.

[15] Kandathil AJ, Kannangai R, Abraham OC, Pulimood SA, Jensen MA, Sridharan G (2009). A comparison of interpretation by three different HIV type 1 genotypic drug resistance algorithms using sequences from non-clade B HIV type 1 strains. *AIDS Research and Human Retroviruses*.

[16] Assoumou L, Houssaïni A, Costagliola D, Flandre P (2010). Relative contributions of baseline patient characteristics and the choice of statistical methods to the variability of genotypic resistance scores: the example of didanosine. *Journal of Antimicrobial Chemotherapy*.

[17] Saigo H, Altmann A, Bogojeska J, Mller F, Nowozin S, Lengauer T (2011). Learning from past treatments and their outcome improves prediction of in vivo response to anti-HIV therapy. *Statistical Applications in Genetics and Molecular Biology*.

[18] van der Laan MJ, Dudoit S (http://www.bepress.com/ucbbiostat/paper130/, 2003). Unified cross-validation methodology for selection among estimators and a general cross-validated adaptive epsilon-net estimator: finite sample oracle inequalities and examples.

[19] van der Laan MJ, Sherri R (2011). *Targeted Learning: Causal Inference for Observational and Experimental Data*.

[20] Sinisi SE, Polley EC, Petersen ML, Rhee SY, van der Laan MJ (2007). Super learning: an application to the prediction of HIV-1 drug resistance. *Statistical Applications in Genetics and Molecular Biology*.

[21] van der Laan MJ, Polley EC, Hubbard AE (2007). *Super Learner*.

[22] Polley EC, van der Laan MJ (2010). *Super Learner in Prediction*.

[23] Rosasco L, de Vito E, Caponnetto A, Piana M, Verri A (2004). Are loss functions all the same?. *Neural Computation*.

[24] Molina JM, Marcelin AG, Pavie J (2005). Didanosine in HIV-1-infected patients experiencing failure of antiretroviral therapy: a randomized placebo-controlled trial. *Journal of Infectious Diseases*.

[25] Marschner IC, Betensky RA, DeGruttola V, Hammer SM, Kuritzkes DR (1999). Clinical trials using HIV-1 RNA-based primary endpoints: statistical analysis and potential biases. *Journal of Acquired Immune Deficiency Syndromes and Human Retrovirology*.

[26] Flandre P, Durier C, Descamps D, Launay O, Joly V (2002). On the use of magnitude of reduction in HIV-1 RNA in clinical trials: statistical analysis and potential biases. *Journal of Acquired Immune Deficiency Syndromes*.

[27] Flandre P, Alcais A, Descamps D, Morand-Joubert L, Joly V (2004). Estimating and comparing reduction in HIV-1 RNA in clinical trials using methods for interval censored data. *Journal of Acquired Immune Deficiency Syndromes*.

[28] Rhee SY, Taylor J, Wadhera G, Ben-Hur A, Brutlag DL, Shafer RW (2006). Genotypic predictors of human immunodeficiency virus type 1 drug resistance. *Proceedings of the National Academy of Sciences of the United States of America*.

[29] Rabinowitz M, Myers L, Banjevic M (2006). Accurate prediction of HIV-1 drug response from the reverse transcriptase and protease amino acid sequences using sparse models created by convex optimization. *Bioinformatics*.

[30] Ruczinski I, Kooperberg C, Leblanc M (2003). Logic regression. *Journal of Computational and Graphical Statistics*.

[31] Sinisi SE, van der Laan MJ (2004). Deletion/substitution/addition algorithm in learning with applications in genomics. *Statistical Applications in Genetics and Molecular Biology*.

[32] Breiman L, Friedman J, Stone C, Olshen RA (1984). *Classification and Regression Trees*.

[33] Breiman L (2001). Random forests. *Machine Learning*.

[34] Prosperi MCF, Altmann A, Rosen-Zvi M (2009). Investigation of expert rule bases, logistic regression, and non-linear machine learning techniques for predicting response to antiretroviral treatment. *Antiviral Therapy*.

[35] Marcelin AG, Flandre P, Pavie J (2004). New genotypic score comprising mutations impacting negatively and positively the virological response to didanosine in treatment-experienced patients from the randomized didanosine add on Jaguar study. *Antiviral Therapy*.

[36] Masquelier B, Assoumou KL, Descamps D (2008). Clinically validated mutation scores for HIV-1 resistance to fosamprenavir/ritonavir. *Journal of Antimicrobial Chemotherapy*.

[37] Vora S, Marcelin AG, Günthard HF (2006). Clinical validation of atazanavir/ritonavir genotypic resistance score in protease inhibitor-experienced patients. *AIDS*.

[38] Flandre P, Marcelin AG, Pavie J (2005). Comparison of tests and procedures to build clinically relevant genotypic scores: application to the Jaguar study. *Antiviral Therapy*.

[39] DiRienzo AG, DeGruttola V, Larder B, Hertogs K (2003). Non-parametric methods to predict HIV drug susceptibility phenotype from genotype. *Statistics in Medicine*.

